# National burden of rheumatoid arthritis in Canada, 1990–2019: findings from the Global Burden of Disease Study 2019 – a GBD collaborator-led study

**DOI:** 10.1136/rmdopen-2023-003533

**Published:** 2024-01-12

**Authors:** Nejat Hassen, Diane Lacaille, Alice Xu, Amani Alandejani, Sophia Sidi, Marjan Mansourian, Zahid A Butt, Leah E Cahill, Ihoghosa Osamuyi Iyamu, Justin J Lang, Juwel Rana, Ranjani Somayaji, Nizal Sarrafzadegan, Jacek A Kopec

**Affiliations:** 1 Arthritis Research Canada, Vancouver, British Columbia, Canada; 2 School of Population and Public Health, The University of British Columbia, Vancouver, British Columbia, Canada; 3 Department of Medicine, The University of British Columbia, Vancouver, British Columbia, Canada; 4 Pediatric Cardiovascular Research Center, Cardiovascular Research Institute, Isfahan University of Medical Sciences, Isfahan, Iran; 5 School of Public Health and Health Systems, University of Waterloo, Waterloo, Ontario, Canada; 6 Al Shifa School of Public Health, Al-Shifa Trust Eye Hospital Rawalpindi, Rawalpindi, Pakistan; 7 Department of Medicine, Dalhousie University, Halifax, Nova Scotia, Canada; 8 Department of Nutrition, Harvard University, Boston, Massachusetts, USA; 9 Knowledge Translation Program, Centre for Health Evaluation and Outcome Sciences, Vancouver, British Columbia, Canada; 10 Centre for Surveillance and Applied Research, Public Health Agency of Canada, Ottawa, Ontario, Canada; 11 Department of Epidemiology, Biostatistics and Occupational Health, McGill University, Montreal, Quebec, Canada; 12 Research and Innovation Division, South Asian Institute for Social Transformation (SAIST), Dhaka, Bangladesh; 13 Department of Family Medicine, University of Calgary, Calgary, Alberta, Canada; 14 Department of Medicine, University of Washington, Seattle, Washington, USA; 15 Isfahan Cardiovascular Research Institute, Cardiovascular Research Institute, Isfahan University of Medical Sciences, Isfahan, Iran; 1 School of Public Health, University of Montreal, Montreal, Quebec, Canada; 2 Department of Community Medicine, Kathmandu University, Kathmandu University, Devdaha, Nepal; 3 Department of Community Health and Epidemiology, University of Saskatchewan, Saskatoon, Saskatchewan, Canada; 4 Department of Public Health, Federal Ministry of Health, Abuja, Nigeria; 5 Department of Pharmaceutical Sciences, The University of British Columbia, Vancouver, British Columbia, Canada; 6 Department of Community Health and Epidemiology, Dalhousie University, Halifax, Nova Scotia, Canada; 7 Department of Health Sciences, Zayed University, Dubai, UAE; 8 Centre for Global Child Health, University of Toronto, Toronto, Ontario, Canada; 9 Centre of Excellence in Women & Child Health, Aga Khan University, Karachi, Pakistan; 10 Office of Institutional Analysis, University of Windsor, Windsor, Ontario, Canada; 11 Department of Neurobiology, Karolinska Institute, Stockholm, Sweden; 12 Division of Neurology, University of Ottawa, Ottawa, Ontario, Canada; 13 Faculty of Kinesiology, University of New Brunswick, Fredericton, New Brunswick, Canada; 14 School of Psychology and Exercise Science, Murdoch University, Murdoch, Western Australia, Australia; 15 Centre for Health Services and Policy Research, The University of British Columbia, Vancouver, British Columbia, Canada; 16 Department of Demography, University of Montreal, Montreal, Quebec, Canada; 17 Department of Social and Preventive Medicine, University of Montreal, Montreal, Quebec, Canada; 18 Faculty of Health and Life Sciences, Coventry University, Coventry, UK; 19 Department of Medicine, McMaster University, Hamilton, Ontario, Canada; 20 Department of Experimental Medicine, McGill University, Montreal, Quebec, Canada; 21 Institute for Health Metrics and Evaluation, University of Washington, Seattle, Washington, USA; 22 Department of Health Metrics Sciences, School of Medicine, University of Washington, Seattle, Washington, USA; 23 Department of Psychiatry and Behavioural Neurosciences, McMaster University, Hamilton, Ontario, Canada; 24 Department of Psychiatry, University of Lagos, Lagos, Nigeria; 25 Department of Medicine, University of Alberta, Edmonton, Alberta, Canada; 26 Population Health Research Institute, McMaster University, Hamilton, Ontario, Canada; 27 Cancer Epidemiology and Prevention Research, Alberta Health Services, Calgary, Alberta, Canada; 28 Department of Oncology, University of Calgary, Calgary, Alberta, Canada; 29 School of International Development and Global Studies, Faculty of Social Sciences, University of Ottawa, Ottawa, Ontario, Canada; 30 The George Institute for Global Health, Imperial College London, London, UK

**Keywords:** Arthritis, Rheumatoid, Epidemiology, Arthritis

## Abstract

**Objective:**

The objectives of this study were: (1) to describe burden of rheumatoid arthritis (RA) and trends from 1990 to 2019 using the Global Burden of Diseases, Injuries and Risk Factors Study (GBD) data, (2) to describe age and sex differences in RA and (3) to compare Canada’s RA burden to that of other countries.

**Methods:**

Disease burden indicators included prevalence, mortality, years of life lost (YLLs), years lived with disability (YLDs) and disability-adjusted life-years (DALYs). GBD estimated fatal and non-fatal outcomes using published literature, survey data and health insurance claims. Data were analysed by Bayesian meta-regression, cause of death ensemble model and other statistical methods. DALYs for Canada were compared with DALYs of countries with similarly high Socio-Demographic Index values.

**Results:**

In Canada, the RA prevalence rate increased by 27% between 1990 and 2019, mortality rate decreased by 27%, YLL rate decreased by 30%, YLD increased by 27% and DALY rate increased by 13%, all age standardised. The decline in RA mortality and YLL rates was especially pronounced after 2002. The disease burden was higher in females for all indicators, and DALY rates were higher among older age groups, peaking at age 75–79 years. Prevalence and DALYs were higher in Canada compared with global rates.

**Conclusion:**

Trends in RA burden indicators over time and differences by age and sex have important implications for Canadian policy-makers, researchers and care providers. Early identification and management of RA in women may help reduce the overall burden of RA in Canada.

**WHAT IS ALREADY KNOWN ON THIS TOPIC**

- There have been regional attempts to characterise the burden of rheumatoid arthritis (RA) in Canada, but no pan-Canadian study has comprehensively summarised the national burden of RA to date.

**WHAT THIS STUDY ADDS**

- This study is the first of its kind to provide a comprehensive overview of RA burden and its trends in Canada. Between 1990 and 2019, RA prevalence and disability-adjusted life-years rates increased, while mortality decreased in Canada. The disease burden was higher in females and older age groups. Some disease burden indicators were higher in Canada compared with global rates.

**HOW THIS STUDY MIGHT AFFECT RESEARCH, PRACTICE OR POLICY**

- Having a comprehensive understanding of RA burden trends in Canada can help health decision-makers plan for better prevention and management strategies at a national level.

## Introduction

Rheumatoid arthritis (RA) is a chronic autoimmune disease, which affects 0.5%–1% of the global population.^1–4^ It is three times as common in women than men and is associated with limited physical function, joint tenderness, fatigue and pain. Other major organs, such as the heart and eyes, may also be affected in serious cases. RA can lead to severe disability and lower quality of life. Numerous studies over the last couple of decades have demonstrated the dynamic nature of RA epidemiology, indicating that studying RA burden is becoming an important topic in this field of research.^2 4 5^

There have been regional attempts to characterise the burden of RA in Canada^5 6^; however, no pan-Canadian study has comprehensively summarised the national epidemiology of RA burden to date. Furthermore, RA burden in Canada as related to its social development has not been explored. In the present study, we examined the data from the Global Burden of Diseases, Injuries and Risk Factors Study (GBD 2019). GBD collects, processes and disseminates up-to-date, comprehensive and comparable health data according to disease, geography, time, age and sex.^7 8^ The objective of this study was to describe burden of RA and trends from 1990 to 2019 in Canada using GBD data by age and sex. The present study also considered how the nation’s Socio-Demographic Index (SDI) contributes to disease burden. All indicators were compared with global rates to contextualise Canada’s RA burden and provide a comprehensive analysis. Having a comprehensive understanding of RA burden trends in Canada can help health decision-makers plan for better prevention and management strategies at a national level.

## Methods

We used data from GBD 2019 on RA prevalence, mortality, years of life lost (YLLs), years lived with disability (YLDs) and disability-adjusted life-years (DALYs) in Canada for the years 1990 to 2019, publicly available at https://vizhub.healthdata.org/gbd-compare/.^9^ We presented the burden of disease indicators as counts or rates per 100 000 population (age-standardised rates) and compared our Canadian findings to global findings. The general methodology used for GBD 2019 has been described in previous publications.^10 11^

### Disease definition

RA was defined based on the 1987 American College of Rheumatology criteria.^12^ The International Classification of Diseases-9 and 10 codes for RA are 714.0–714.9 and M05, M06 and M08, respectively.

RA severity was divided into three levels: mild, moderate and severe. Each level is associated with a disability weight (DW): 0.117 (95% uncertainty interval (UI) 0.080 to 0.163) for mild RA, 0.317 (95% UI 0.216 to 0.440) for moderate RA and 0.581 (95% UI 0.403 to 0.739) for severe RA. DWs were derived from large population studies in several countries and represent the amount of health lost due to RA on a scale from 0 (no disability) to 1 (death).^13^ The severity distribution was derived from an analysis of the Medical Expenditure Panel Surveys in the USA. Details on this process are available in previous GBD publications.^7 8 11^

### Measures of disease burden

Age-standardised and all-age data for all age groups and both sexes were collected. Mortality and prevalence were estimated using vital statistics, published literature, survey and surveillance data, and health insurance claims. YLLs measure premature death calculated as RA-related deaths in each age group multiplied by the standard life expectancy at that age. The standard life expectancy was taken from the lowest observed risk of death for each 5-year age group in all populations greater than 5 million. YLDs reflect the amount of time in a year that people live with any short-term or long-term health condition, considering the severity of that condition. They were calculated by multiplying RA prevalence by DWs for each severity level, age, sex and year. DALYs were calculated as the sum of YLLs and YLDs for each age group, sex and year. Finally, SDI combines income per capita, average educational attainment and fertility rates. DALYs for Canada were compared with DALYs of countries with the same high-SDI classification as a group. Calculation details of disease burden indicators can be found in previous studies.^14–16^

### Data sources

GBD conducted a systematic review of global RA prevalence using Ovid MEDLINE, EMBASE, CINAHL, CAB abstracts, WHOLIS and SIGLE databases.^7^ Population surveys and scientific literature from previous GBD iterations were added when available. All data collected were stratified by age and sex. Data were included only if they met the following criteria: population-based studies, representative of the national or regional population, primary data sources, sample size greater than 150, non-review papers and focused specifically on RA.

Canada-specific RA data were obtained from Canada Vital Records and an epidemiological study conducted in the province of Ontario.^5^ Vital statistics were used to estimate annual RA-specific and all-cause mortality during the study period. Population-based data from the Ontario Rheumatoid Arthritis Administrative Database (ORAD) had data available for all Ontarians with RA. The ORAD records were linked with census data to calculate prevalence from 1996 to 2010. National census data and the Ontario Registered Persons Database were used for data regarding individuals’ age, sex and vital status.

### Data modelling

Although a case definition based on the ACR 1987 criteria was used as a reference, some data using other definitions were identified in the systematic review. We applied statistical modelling (referred to as ‘cross-walking’) to adjust for different RA definitions. Studies using non-ACR criteria were labelled as a separate covariate in the analysis. Other covariates were created for data that came from administrative health system sources, data that covered regional rather than national populations and for US MarketScan data in 2000.^10^

Pooled data from different sources were used to model and calculate estimates for the burden of disease indicators. DisMod-MR 2.1, a Bayesian meta-regression tool, was used to model non-fatal outcomes, adjust for methodological differences and to ensure consistency. It was assumed RA cases were non-existent before the age of 5 years. DisMod-MR produced a full set of age/sex/region/year-specific estimates for non-fatal burden of disease indicators. The GBD cause of death ensemble model was used to model mortality rates.^7^

## Results

### Prevalence

**Figure 1:**
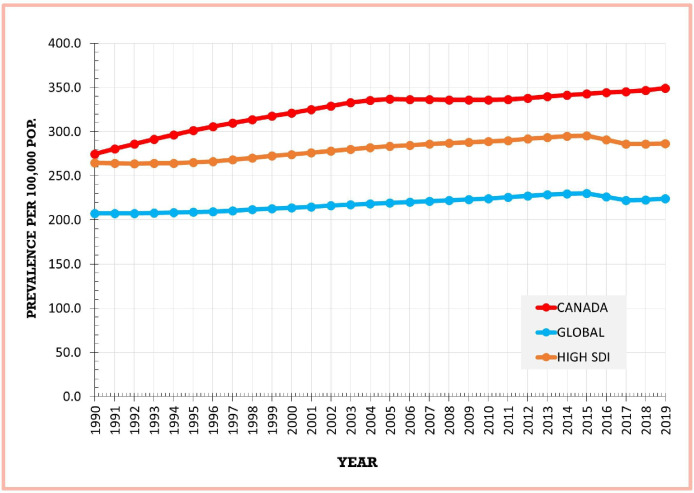
Age-standardised RA prevalence, Canada, global, high SDI, 1990–2019. RA, rheumatoid arthritis; SDI, Socio-Demographic Index.

The age-standardised prevalence rate for Canada increased by 27.0% from 274.8 (269.5–280.1) per 100 000 in 1990 to 349.1 (342.5–356.0) in 2019 (table 1). The age-standardised prevalence rate was substantially higher in females compared with males. When stratified by sex, the age-standardised rates increased by 26.0% in males and 29.0% in females. Globally, the age-standardised prevalence rate increased by 8.0% from 207.5 (190.0–227.0) per 100 000 in 1990 to 224.25 (204.9–246.0) in 2019 (online supplemental table S2). Global rates remained lower than Canadian rates from 1990 to 2019. Prevalence for high SDI countries as a group increased by 8.0% (online supplemental table S3) and was lower than Canadian rates, but higher than global rates throughout the study period (figure 1).

10.1136/rmdopen-2023-003533.supp1 Supplementary data

**Table 1:** Change over time for all-age and age-standardised burden of disease indicators per 100 000, Canada, 1990–2019

Indicator (95%UI)	Standardisation	Both (female and male)		
		Year		Change
		1990	2019	
Prevalence	All age	317.0 (310.7 to 323.3)	543.1 (532.1 to 553.6)	71%
	Age standardised	274.8 (269.5 to 280.1)	349.1 (342.5 to 356.0)	27%
Mortality	All age	0.7 (0.4 to 0.9)	0.9 (0.5 to 1.1)	22%
	Age standardised	0.6 (0.4 to 0.7)	0.4 (0.3 to 0.6)	−27%
YLLs	All age	13.4 (8.3 to 16.3)	14.5 (8.7 to 17.7)	8%
	Age standardised	11.1 (6.9 to 13.7)	7.8 (4.8 to 9.4)	−30%
YLDs	All age	41.7 (28.9 to 56.1)	70.6 (47.9 to 94.5)	69%
	Age standardised	36.2 (25.1 to 48.9)	45.9 (31.6 to 61.6)	27%
DALYs	All age	55.1 (41.2 to 69.7)	85.1 (62.6 to 109.5)	54%
	Age standardised	47.3 (35.3 to 60.1)	53.7 (39.2 to 69.4)	13%
Indicator (95%UI)	Standardisation	Female		
		Year		Change
		1990	2019	
Prevalence	All age	461.3 (450.6 to 470.9)	783.0 (764.4 to 801.5)	70%
	Age standardised	382.8 (373.9 to 390.9)	493.5 (482.8 to 504.6)	29%
Mortality	All-age	1.1 (0.5 to 1.4)	1.2 (0.6 to 1.6)	14%
	Age standardised	0.8 (0.4 to 1.0)	0.6 (0.3 to 0.7)	−28%
YLLs	All age	19.2 (10.1 to 25.4)	19.1 (9.4 to 24.7)	0%
	Age standardised	14.4 (7.7 to 19.3)	9.7 (5.1 to 12.6)	−33%
YLDs	All age	60.3 (42.0 to 81.1)	101.4 (69.1 to 135.8)	68%
	Age standardised	50.4 (35.1 to 67.5)	64.7 (44.8 to 86.4)	29%
DALYs	All age	79.5 (59.0 to 100.9)	120.5 (87.2 to 156.0)	52%
	Age standardised	64.7 (48.2 to 82.1)	74.5 (53.6 to 96.6)	15%
Indicator (95%UI)	Standardisation	Male		
		Year		Change
		1990	2019	
Prevalence	All age	168.9 (166.0 to 172.0)	295.4 (290.2 to 300.6)	75%
	Age standardised	154.7 (152.2 to 157.5)	195.1 (191.8 to 198.3)	26%
Mortality	All age	0.4 (0.2 to 0.4)	0.6 (0.3 to 0.7)	46%
	Age standardised	0.4 (0.2 to 0.4)	0.3 (0.2 to 0.4)	−18%
YLLs	All age	7.4 (4.4 to 8.7)	9.7 (5.8 to 11.7)	30%
	Age standardised	6.9 (4.1 to 8.1)	5.5 (3.4 to 6.7)	−20%
YLDs	All age	22.5 (15.2 to 30.8)	38.8 (26.1 to 51.6)	72%
	Age standardised	20.6 (14.0 to 28.1)	25.9 (17.5 to 34.8)	26%
DALYs	All age	30.0 (22.3 to 38.2)	48.5 (35.5 to 61.9)	62%
	Age standardised	27.5 (20.7 to 35.2)	31.4 (22.7 to 40.4)	14%

DALYs, disability-adjusted life years; UI, uncertainty intervals; YLDs, years lived with disability; YLLs, years of life lost.

### Mortality and YLLs

**Figure 2:**
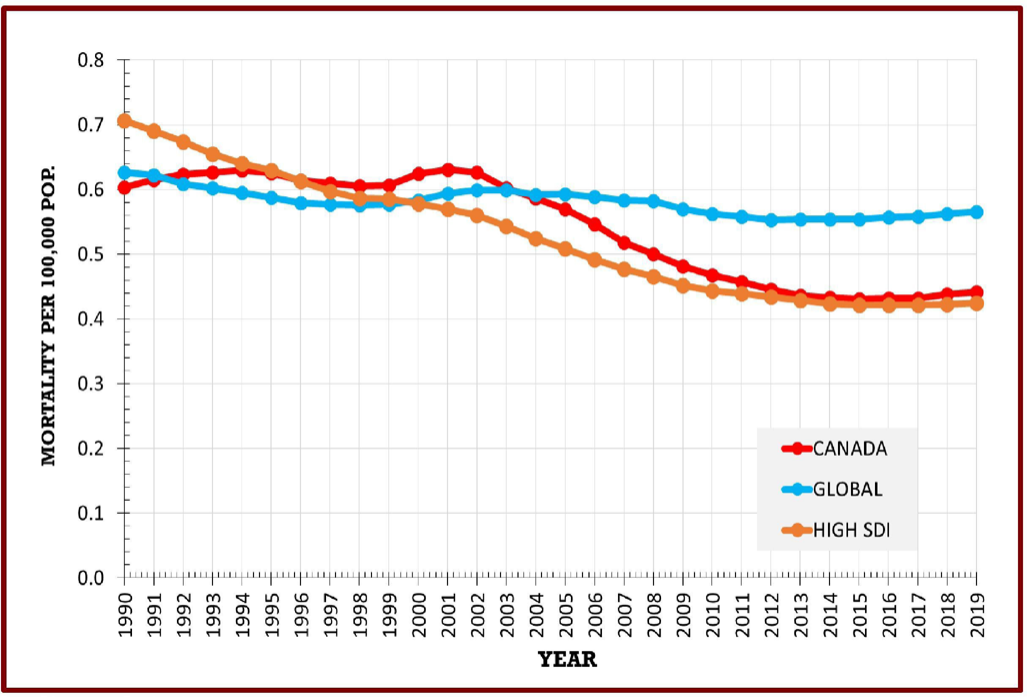
Age-standardised RA mortality, Canada, global, high SDI, 1990–2019. RA, rheumatoid arthritis; SDI, Socio-Demographic Index.

The age-standardised mortality rate decreased by 27.0% from 0.6 (0.4–0.7) per 100 000 in 1990 to 0.4 (0.3–0.6) in 2019 (table 1). Mortality declined more in females than in males (28.0% vs 18.0%) but remained substantially higher in females. The global age-standardised mortality rate decreased by 10.0% from 0.6 (0.5–0.8) per 100 000 in 1990 to 0.6 (0.4–0.7) in 2019 (online supplemental table S2). Mortality in Canada started to see a steeper decline compared with the global rate post-2002 (figure 2). Mortality for high SDI countries decreased by 40.0% over the study period (figure 2, online supplemental table S3).

The YLL rates in Canada followed a pattern similar to mortality. The age-standardised YLL rate decreased by 30.0% from 1990 to 2019, 33.0% for females and 20.0% for males (table 1). The global trend showed a 15.0% decrease from 12.0 (9.6–14.8) per 100 000 in 1990 to 10.2 (7.9–11.9) in 2019 (online supplemental table S2). Compared with the global trend, Canada saw a sharper decline post-2002, mirroring the trend for mortality in the country (online supplemental figure S1). YLLs for high SDI countries decreased by 42.0% and followed similar trends as Canada (online supplemental table S3).

### YLDs and DALYs

The age-standardised YLD rate in Canada increased by 27.0%, from 36.2 (25.1–48.9) per 100 000 in 1990 to 45.9 (31.6–61.6) in 2019 (table 1). This rate increased by 29.0% and 26.0% for females and males, respectively. Canada’s YLD rate increase was higher compared with global rate from 1990 to 2019. Globally, the age-standardised YLD rate increased by 8% from 27.1 (18.8–36.4) per 100 000 in 1990 to 29.4 (20.3–39.5) in 2019 (online supplemental table S2 and figure S2). Age-standardised YLD rates for high SDI countries also increased by 8.0% during the same period (online supplemental table S3).

**Figure 3:**
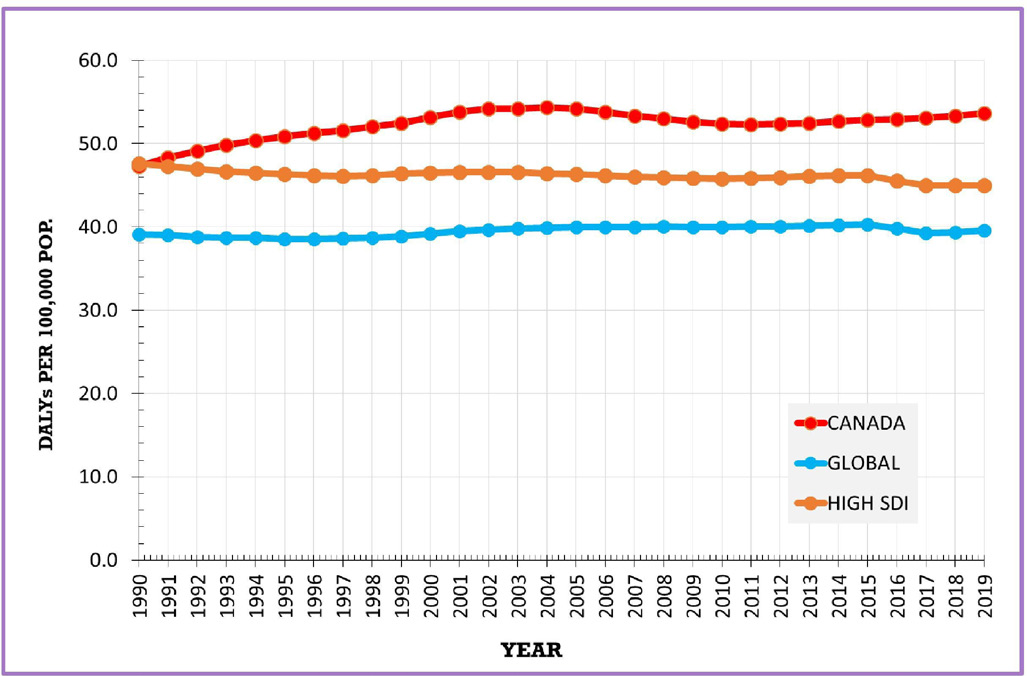
Age-standardised RA DALYs, Canada, global, high SDI, 1990–2019. DALYs, disability-adjusted life-years; RA, rheumatoid arthritis; SDI, Socio-Demographic Index.

DALYs in Canada increased at a slower pace than YLDs. The age-standardised DALY rate increased by 13.0%, from 47.3 (35.3–60.1) per 100 000 in 1990 to 53.7 (39.2–69.4) in 2019 (table 1). The rate increased by 15.0% and 14.0% for females and males, respectively (table 1). Compared with the Canadian rate, the global DALYs rate was substantially lower and remained stable during the study period (figure 3). DALYs for high SDI countries also remained relatively stable, decreasing by 5.0% during the same period (online supplemental table S3). There was a weak but statistically significant correlation (R=0.16) between SDI and DALY rates based on data for 204 countries (online supplemental figure S3).

**Figure 4:**
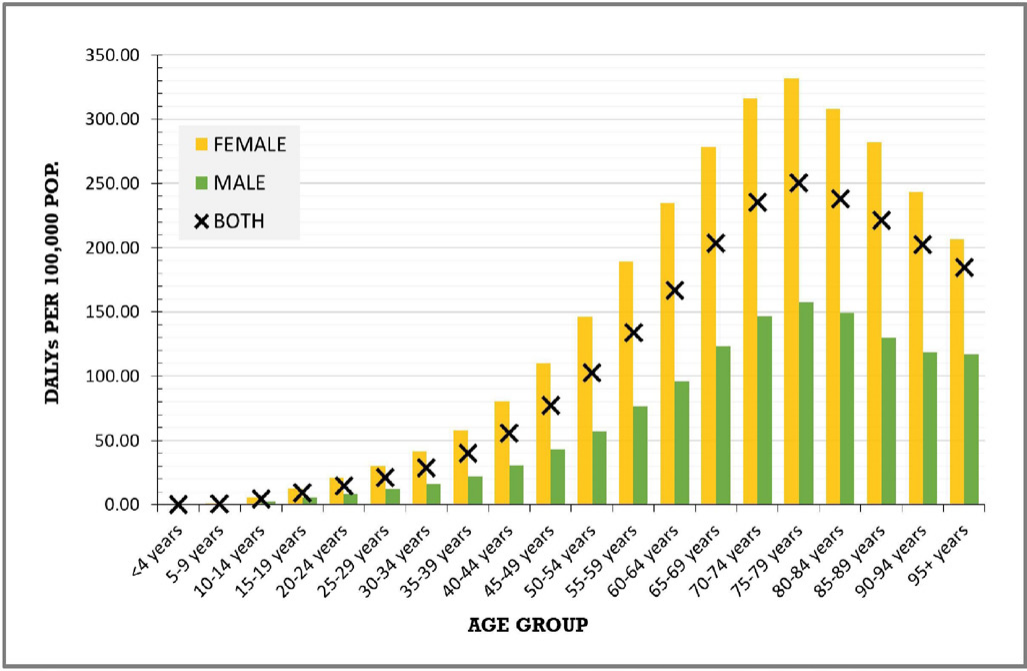
RA DALYs by age group and sex, Canada, 2019. DALYs: disability-adjusted life-years; RA, rheumatoid arthritis.

The highest DALY rates were observed in the 75–79 and 70–74 years age groups, with rates of 250.6 (188.1–322.3) per 100 000 and 238.0 (176.0–304.7), respectively, in the year 2019. During the same year, females accounted for nearly two-thirds of the DALYs from RA across all age groups (figure 4).

**Figure 5:**
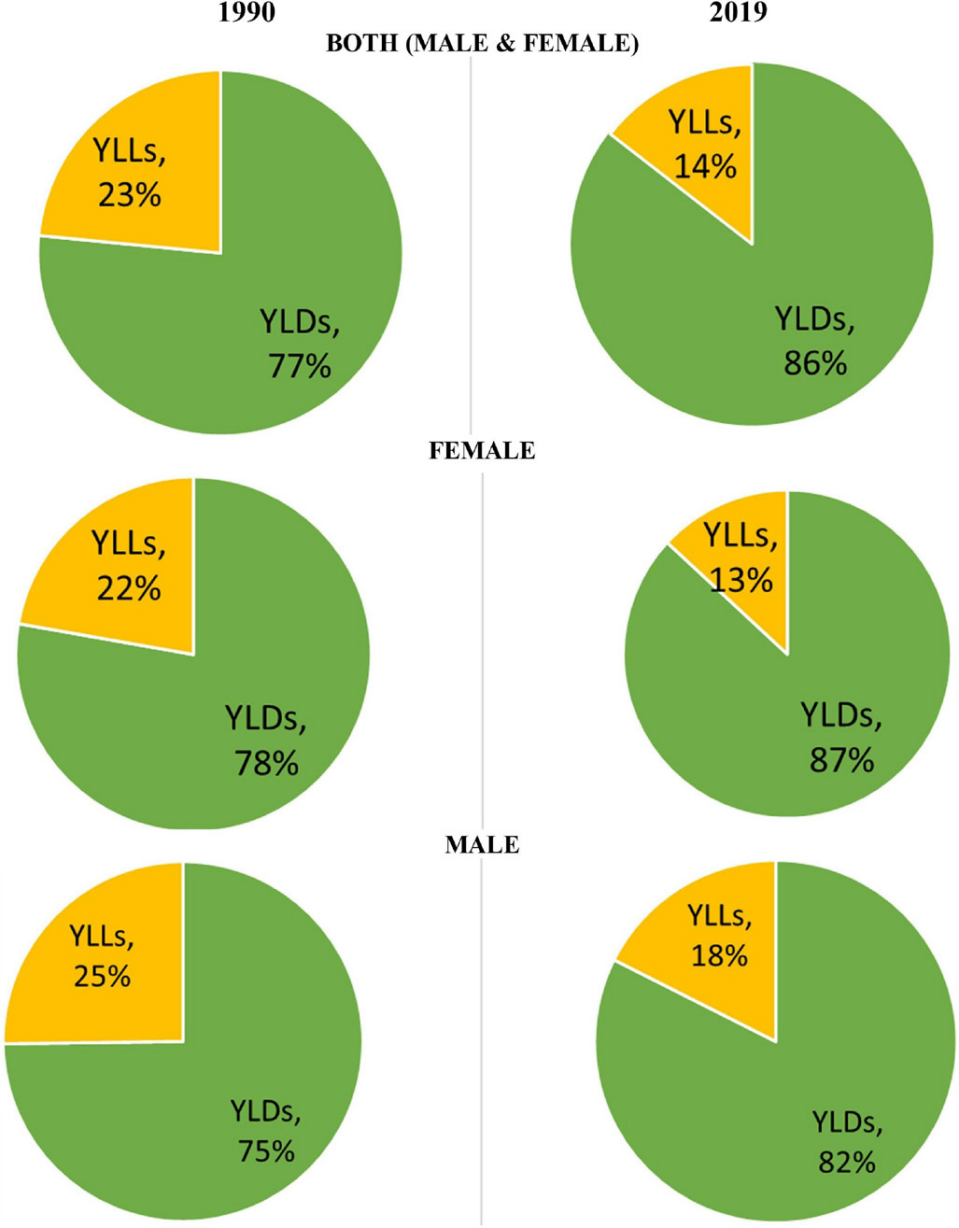
Proportion of age-standardised RA DALYs due to age-standardised YLLs and age-standardised YLDs, Canada, 1990 vs 2019. RA, rheumatoid arthritis; YLDs, years lived with disability; YLLs, years of life lost.

The proportion of YLLs and YLDs contributing to DALYs changed considerably over time. In 1990, close to a quarter (23.0%) of DALYs was made up of YLLs and 77.0% of YLDs. In 2019, YLDs contributed 86.0% of all DALYs in Canada (figure 5).

## Discussion

In this paper, we present data on the prevalence, mortality, YLL, YLD and DALY rates for RA in Canada from 1990 to 2019. In 2019, there were 198 326 prevalent cases, 325 deaths, 5283 YLLs, 25 782 YLDs and 31 065 DALYs due to RA in Canada. The number of people living with RA increased by 130.0% between 1990 and 2019. This increase was due to population growth, population ageing and an increase in age-specific prevalence rates. Age-standardised prevalence rates increased over time and were significantly greater in females. Age-standardised mortality and YLLs mirrored each other’s declining rates for both sexes. Age-standardised YLDs and DALYs followed increasing trends with time, although the trend for YLDs was more pronounced than for DALYs. DALY rates were higher in females and peaked at age 75–79 years for both females and males.

Previous studies have indicated that RA prevalence is higher in females, with a female to male ratio of 3:1^17^; a slightly lower ratio was noted in the present study. Furthermore, studies from the UK and USA confirm the difference between male and female prevalence rates seen in the present study.^3 18^ In a multinational cross-sectional study consisting of 25 countries, females were identified to have poorer disease activity scores compared with males, including swollen joint count, tender joints, fatigue and erosion.^19^ Generally, females report more severe symptoms and greater disability compared with their male counterparts,^19^ perhaps reflective of gender biases in patient care that lead to poorer health outcomes in women.^20^ Studies suggest that males have better responses to biological treatments, and that male gender is a significant predictor of remission in early RA disease stage.^19 21^ The reasons for greater remission rates in males are unclear and warrant further investigation. Higher prevalence in later years may also be due to increased awareness about RA among patients and physicians, and increased testing for RA by physicians, thus detection bias must be considered as well. The number of people with RA will also increase with population ageing, which is a consequence of higher RA rates in older populations and a reduction in RA mortality due to improved medical treatments and better disease management.

Genetic differences have been suggested to explain the predominance of RA in women. X-chromosome’s abnormalities are associated with several autoimmune diseases and have been postulated to contribute to the pathogenesis of RA.^17 22^ Furthermore, X-linked genetic mutations lead to deficient antibody production or overactive immune systems.^22^ Lastly, oestrogen hormone levels have been identified to play a role in downregulating inflammatory immune responses.^23^ During pregnancy, when oestrogen levels increase, disease activity is overturned in up to 75% of women, only to return as flare-ups post partum.^24^ Hence, oral contraceptives and hormone replacement therapy may have protective features against RA disease activity.^25^

Notably, the age-standardised mortality and YLL rates significantly declined after 2002, while the YLDs increased in Canada. These trends might be related to changes in RA treatment practices. Joint destruction in RA begins early in the course of the disease. Systemic inflammation causes inflammatory cytokines to promote bone resorption and prevent bone formation, producing a net bone loss.^26 27^ Some control can be achieved with non-steroidal anti-inflammatory drugs or glucocorticoids. However, recent RA management strategies underscore the importance of early, appropriate and aggressive treatment to reduce the risk of further joint damage as much as possible.^28^ Early, appropriate and aggressive treatment (treat-to-target approach) is recommended to minimise the risk of joint damage consequential of RA.^29^ The use of disease-modifying antirheumatic drugs (DMARDs) has become the basis of therapy and are prescribed early following RA diagnosis. Traditional DMARDs, such as methotrexate, re-establish normal bone formation processes and promote disease remission.^26^ Methotrexate is also associated with reduced cardiovascular mortality in RA. Cardiovascular disease is the most prevalent complication in patients with RA and remains a significant contributor to excess mortality.^6 30^ Methotrexate has been associated with reduced systemic inflammation, which may be the mechanism by which it reduces cardiovascular mortality.^31^ Methotrexate is often the initial DMARD used due to its efficacy, low cost, minimal side effects and demonstration of slowing down radiographic disease progression.^28^ Early initiation of DMARDs is important for better clinical outcomes and prevention of erosive disease.^29 32^

In addition to traditional DMARDs, new biological DMARDs that target specific inflammation mechanisms were introduced in the late 1990s/early 2000s.^33^ Etanercept, commonly known as Enbrel, was first globally introduced in 1998 for moderate to severe RA symptoms in the USA.^34^ In Canada, Enbrel injections were approved to be used in conjunction with methotrexate pills and have been marketed nationally since March 2001.^35^ This new treatment strategy, along with improved technology to detect RA earlier, has been shown to alter the clinical course of RA and slow disease progression to the point of remission.^36^ As a result, patients may live longer with the disease, although with some disability. This may be one reason for the increase in RA prevalence and YLDs. Furthermore, patients may live longer with RA due to the protective effect of the newly implemented biologics against the occurrence of RA complications and, consequently, RA-related mortality.^37^

There are some differences in mortality and YLLs between Canada and global rates. While these rates were comparable between 1990 and 2000, the Canadian rates dropped dramatically after 2002 while the global rates remained relatively the same. This may be indicative of Canada’s early introduction and use of new biological DMARDs and using an aggressive treat-to-target approach. While implementing the treat-to-target approach is recommended to controlling RA to reach remission, not all countries may have the proper healthcare infrastructure to do so. Financial constraints in some countries can result in the unaffordability or unavailability of RA medications such as DMARDs. This can hinder access to effective treatment options. Furthermore, shortage of rheumatologists and specialised healthcare centres can delay treatment and lead to poorer clinical outcomes.^38^ Differences in how biologics and treat-to-target treatments approaches were first implemented may contribute to differences in Canada and global mortality rates. However, the exact cause for these discrepancies warrants further investigation.

Moreover, comparing RA-related DALYs according to socioeconomic development is rarely considered in epidemiological studies. Canada is a country with one of the highest SDIs in the world and Canada’s DALY rate was similar to the average rate for the high SDI countries in 1990. However, between 1990 and 2019, the rate increased in Canada and decreased in high SDI countries as a group. This divergence of trends was mainly due to different trends in RA prevalence, whereas mortality trends were more similar, especially in the last two decades. The reasons for a faster increase in RA prevalence in Canada compared with countries with a similar SDI in GBD data require further study.

While the exact cause of RA is unknown, there are several risk factors that may increase its prevalence. It is thought that RA prevalence in first-degree relatives accounts for 20%–50% of RA cases, with a greater proportion contributing to seropositive RA.^39^ Genetic predispositions to inflammation may also lead to abnormalities in several inflammatory pathways, predisposing patients to developing autoantibodies.^40^ However, smoking is the only GBD-established risk factor associated with RA,^8^ responsible for about 14% of all RA-related DALYs in Canada. It is possible that smoking may lead to a possible increase in the prevalence of RA. Numerous studies have identified a strong association between smoking and RA.^41–43^ It has been estimated that in some countries exposure to smoking accounts for 20%–30% of RA cases.^44^ This association is more significant in men and individuals who have smoked for over 20 years. Other modifiable risk factors such as obesity and dietary behaviours such as consuming diets high in red meat, sugar, omega-3 fatty acids and caffeine, and low in antioxidants have been studied in literature.^45–49^ However, these are not reflected in our results because the data on their association with RA are inconclusive. Further studies on these factors are needed, especially because mitigating these modifiable risk factors can potentially establish preventative interventions for RA development.

### Strengths and limitations

Monitoring trends in RA burden is important for informing public policy. This study is the first of its kind to provide a comprehensive overview of RA burden and its trends in Canada. Knowing the burden can help health agencies better understand the situation and strategise targeted public health solutions to manage the disease nationwide, potentially modifying the national approach to managing RA. For communities, this study may help inform education dissemination strategies to inform the public about the effect of RA on Canadian patients’ lives. The study also lays the foundation for future researchers to investigate gaps about aspects of RA burden in Canada and how to appropriately address them.

A notable strength of this study is its comprehensiveness, as it analyses data for the greatest number of RA burden indicators for a single nation to date. This study also uses data retrieved from multiple sources including Canadian vital statistics and epidemiological literature. Sophisticated data processing and modelling methods used by GBD are also a strength. Because the estimates are corrected for differences in RA definitions across studies, we were able to compare Canada with other countries and evaluate changes in RA burden over time.

A limitation of this study is that global GBD RA data are retrieved from a scarce number of countries.^7^ Another limitation is the lack of known predictors of RA burden, which affected the ability of statistical models to estimate burden for countries with sparse or non-existent data, including Canada. Furthermore, the present study assumed that disease severity distributions were the same in males and females and remained stable over time. Some studies suggest that females tend to have more severe disease than males.^19 21^ There is also evidence of modern treatments being more effective in reducing RA-related disability.^50^ Another important limitation of this study remains the lack of access to regional data from multiple provinces in Canada. Data used to estimate RA prevalence trends in Canada were largely derived from an epidemiological study in a single province.^5^ That study, published in 2014, may not sufficiently reflect the recent trends in RA prevalence in Canada. Other limitations to this study may inherently affect our estimates. Finally, the only risk factor for RA in GBD is smoking. More data on risk factors for RA would have contributed to a more comprehensive overview of the RA burden in Canada.

## Conclusion

RA is a major public health challenge that affects a considerable portion of the Canadian population. Age-standardised prevalence, YLDs and DALYs increased while mortality and YLLs decreased between 1990 and 2019. Rates for all indicators were higher in females than males. The Canadian healthcare system should prioritise early identification and disease management interventions, especially for females, to reduce the overall burden of RA. Minimal information on RA risk factors (except smoking) limits us in offering wide-ranging disease prevention recommendations. Large-scale, population-based studies to identify significant risk factors for RA are needed. Moreover, availability of data from multiple provincial RA databases would increase the accuracy and generalisability of our estimates for Canada. We hope that the present study provides a comprehensive overview of RA disease burden in Canada that will be useful to researchers, care providers and policy-makers.

## Data Availability

Data are available in a public, open access repository. Data are available in a public, open access repository at https://vizhub.healthdata.org/gbd-compare/
