# Supplementary material for: National burden of rheumatoid arthritis in Canada, 1990–2019: findings from the Global Burden of Disease Study 2019 – a GBD collaborator-led study
Source: RMD Open. 2024 Jan 12;10(1):e003533. doi: 10.1136/rmdopen-2023-003533 (PMC10806499; doi:10.1136/rmdopen-2023-003533)
Supplement: Supplementary data [file rmdopen-2023-003533supp001.pdf]

BMJ Publishing Group Limited (BMJ) disclaims all liability and responsibility arising from any reliance placed on this supplemental material which has been supplied by the author(s)

Table S1. Change over time for burden of disease indicators, count, Canada, global, 1990-2019

Indicator (95%UI)	Location	BOTH (female & male) YEAR 1990	2019	Change
Prevalence	Canada	86,413.6 (84,703.0-88,119.6)	198,325.8 (194,316.1-202,185.0)	**130%**
	Global	9,019,524.2 (8,239,786.0-9,898,679.4)	18,583,481.1 (16,955,383.3-20,433,859.1)	**106%**
Mortality	Canada	198.2 (119.5-238.9)	324.7 (177.5-408.9)	**64%**
	Global	22,191.7 (17,533.6-27,197.5)	44,408.4 (33,953.7-51,747.8)	**100%**
YLLs	Canada	3647.6 (2267.0-4455.0)	5282.8 (3178.6-6473.3)	**45%**
	Global	475,759.6 (381,207.9-580,910.6)	829,549.2 (640,851.6-968,237.4)	**74%**
YLDs	Canada	11,360.4 (7875.1-15,296.0)	25,782.2 (17,492.9-34,492.2)	**127%**
	Global	1,186,862.3 (821,205.8-1,596,156.9)	2,433,039.9 (1,681,353.6-3,276,916.0)	**105%**
DALYs	Canada	15,008.0 (11,227.5-19,002.7)	31,065.0 (22,873.4-39,974.3)	**107%**
	Global	1,662,622.0 (1,273,128.9-2,077,289.6)	3,262,589.1 (2,510,208.4-4,091,554.9)	**96%**
**Indicator (95%UI)**	**Location**	**FEMALE YEAR 1990**	**2019**	**Change**
Prevalence	Canada	63,700.2 (62,217.1-65,023.6)	145,243.7 (141,803.6-148,677.0)	**128%**
	Global	6,468,678.3 (5,934,756.1-7,069,464.2)	13,194,101.1 (12,110,684.1-14,447,079.9)	**104%**
Mortality	Canada	147.5 (74.8-195.2)	226.2 (102.3-303.6)	**53%**
	Global	15,420.2 (11,419.5-20,651.5)	30,576.3 (20,414.8-37,673.8)	**98%**
YLLs	Canada	2647.0 (1388.1-3500.6)	3540.0 (1742.0-4575.5)	**34%**
	Global	323,134.7 (238,384.1-433,195.0)	564,427.0 (380,335.0-705,395.9)	**75%**
YLDs	Canada	8332.5 (5793.1-11,191.5)	18,815.3 (12,822.3-25,192.9)	**126%**
	Global	845,490.9 (586,358.5-1,135,198.1)	1,717,349.3 (1,188,996.0-2,306,242.0)	**103%**
DALYs	Canada	10,979.5 (8146.9-13,930.8)	22,355.4 (16,174.4-28,935.2)	**104%**
	Global	1,168,625.6 (875,452.9-1,464,387.9)	2,281,776.3 (1,727,389.4-2,875,227.6)	**95%**
**Indicator (95%UI)**	**Location**	**MALE YEAR 1990**	**2019**	**Change**
Prevalence	Canada	22,713.2 (22,320.2-23,125.8)	53,082.2 (52,150.2-54,022.2)	**134%**
	Global	2,550,845.9 (2,302,459.3-2,819,170.2)	5,389,380.0 (4,883,676.1-5,952,656.0)	**111%**
Mortality	Canada	50.7 (28.5-59.0)	98.6 (54.3-121.5)	**95%**
	Global	6771.6 (5403.6-7489.2)	13,832.1 (11,943.8-15,629.0)	**104%**
YLLs	Canada	1000.6 (597.4-1165.4)	1742.8 (1047.7-2104.4)	**74%**
	Global	152,624.9 (124,227.3-169,324.5)	265,122.3 (232,098.1-300,643.2)	**74%**
YLDs	Canada	3027.9 (2040.8-4146.7)	6966.9 (4687.1-9277.0)	**130%**
	Global	341,371.5 (233,681.6-460,324.2)	715,690.6 (493,796.6-968,783.3)	**110%**
DALYs	Canada	4028.5 (3004.8-5140.1)	8709.7 (6384.0-11,120.4)	**116%**
	Global	493,996.3 (384,815.2-616,199.0)	980,812.8 (759,988.8-1,234,948.7)	**99%**

UI: uncertainty intervals; YLLs: years of life lost, YLDs: years lived with disability; DALYs: disability-adjusted life years

Table S2. Change over time for all-age and age-standardized burden of disease indicators per 100,000, global, 1990-2019

Indicator (95%UI)	Standardization	BOTH (female & male) YEAR 1990	2019	Change
Prevalence	All-age	168.6 (154.0-185.0)	240.2 (219.1-264.1)	**42%**
	Age-standardized	207.5 (190.0-227.0)	224.3 (204.9-246.0)	**8%**
Mortality	All-age	0.4 (0.3-0.5)	0.6 (0.4-0.7)	**40%**
	Age-standardized	0.6 (0.5-0.8)	0.6 (0.4-0.7)	**-10%**
YLLs	All-age	8.9 (7.1-10.9)	10.7 (8.3-12.5)	**21%**
	Age-standardized	12.0 (9.6-14.8)	10.2 (7.9-11.9)	**-15%**
YLDs	All-age	22.2 (15.4-29.8)	31.4 (21.7-42.4)	**42%**
	Age-standardized	27.1 (18.8-36.4)	29.4 (20.3-39.5)	**8%**
DALYs	All-age	31.1 (23.8-38.8)	42.2 (32.4-52.9)	**36%**
	Age-standardized	39.1 (30.1-48.6)	39.6 (30.5-49.5)	**1%**
**Indicator (95%UI)**	**Standardization**	**FEMALE YEAR 1990**	**2019**	**Change**
Prevalence	All-age	243.5 (223.4-266.2)	342.13 (314.03-374.62)	**40%**
	Age-standardized	287.0 (263.6-313.6)	309.16 (283.05-338.68)	**8%**
Mortality	All-age	0.6 (0.4-0.8)	0.79 (0.53-0.98)	**36%**
	Age-standardized	0.8 (0.6-1.0)	0.70 (0.47-0.86)	**-8%**
YLLs	All-age	12.2 (9.0-16.3)	14.64 (9.86-18.29)	**20%**
	Age-standardized	15.0 (11.1-20.1)	12.95 (8.72-16.19)	**-14%**
YLDs	All-age	31.8 (22.1-42.7)	44.53 (30.83-59.80)	**40%**
	Age-standardized	37.4 (26.0-50.2)	40.31 (27.91-54.09)	**8%**
DALYs	All-age	44.0 (33.0-55.1)	59.17 (44.79-74.56)	**34%**
	Age-standardized	52.4 (39.4-65.3)	53.26 (40.20-67.08)	**2%**
**Indicator (95%UI)**	**Standardization**	**MALE YEAR 1990**	**2019**	**Change**
Prevalence	All-age	94.7 (85.5-104.7)	138.9 (125.8-153.4)	**47%**
	Age-standardized	122.4 (111.2-134.7)	134.6 (122.3-148.4)	**10%**
Mortality	All-age	0.3 (0.2-0.3)	0.4 (0.3-0.4)	**44%**
	Age-standardized	0.5 (0.4-0.5)	0.4 (0.4-0.5)	**-11%**
YLLs	All-age	5.7 (4.6-6.3)	6.8 (6.0-7.8)	**20%**
	Age-standardized	8.5 (6.8-9.4)	7.1 (6.2-8.1)	**-16%**
YLDs	All-age	12.7 (8.7-17.1)	18.4 (12.7-24.9)	**46%**
	Age-standardized	16.2 (11.1-21.8)	17.8 (12.3-24.1)	**10%**
DALYs	All-age	18.3 (14.3-22.9)	25.3 (19.6-31.8)	**38%**
	Age-standardized	24.7 (19.5-30.6)	24.9 (19.4-31.3)	**1%**

UI: uncertainty intervals; YLLs: years of life lost, YLDs: years lived with disability; DALYs: disability-adjusted life years

BMJ Publishing Group Limited (BMJ) disclaims all liability and responsibility arising from any reliance placed on this supplemental material which has been supplied by the author(s)

Table S3. Change over time for all-age and age-standardized burden of disease indicators per 100,000, high SDI countries, 1990-2019

Indicator (95%UI)	Standardization	BOTH (female & male) YEAR 1990	2019	Change
Prevalence	All-age	315.6 (292.8-341.2)	432.0 (399.8-467.7)	**37%**
	Age-standardized	264.7 (245.3-286.9)	286.7 (265.2-311.8)	**8%**
Mortality	All-age	0.9 (0.6-1.2)	0.9 (0.6-1.2)	**-6%**
	Age-standardized	0.7 (0.5-0.9)	0.4 (0.3-0.6)	**-41%**
YLLs	All-age	16.6 (11.0-21.8)	14.0 (10.4-19.2)	**-16%**
	Age-standardized	13.1 (8.6-17.1)	7.6 (5.7-10.6)	**-42%**
YLDs	All-age	41.0 (28.8-54.7)	55.7 (38.7-74.6)	**36%**
	Age-standardized	34.6 (24.2-46.2)	37.4 (25.8-50.3)	**8%**
DALYs	All-age	57.6 (44.1-72.1)	69.7 (51.8-88.5)	**21%**
	Age-standardized	47.6 (36.3-59.7)	45.0 (33.3-57.7)	**-5%**
**Indicator (95%UI)**	**Standardization**	**FEMALE YEAR 1990**	**2019**	**Change**
Prevalence	All-age	468.3 (436.4-504.0)	633.5 (589.5-685.3)	**35%**
	Age-standardized	373.0 (346.7-403.7)	413.8 (383.2-450.2)	**11%**
Mortality	All-age	1.4 (0.8-2.0)	1.2 (0.7-1.7)	**-12%**
	Age-standardized	0.9 (0.5-1.3)	0.5 (0.3-0.8)	**-40%**
YLLs	All-age	24.3 (13.9-34.2)	19.3 (12.2-29.1)	**-21%**
	Age-standardized	16.8 (9.7-23.7)	9.7 (6.4-15.1)	**-42%**
YLDs	All-age	60.5 (42.6-80.2)	81.3 (56.6-108.8)	**34%**
	Age-standardized	48.6 (34.1-64.6)	53.9 (37.4-72.4)	**11%**
DALYs	All-age	84.9 (63.8-106.9)	100.6 (74.3-128.1)	**19%**
	Age-standardized	65.5 (48.9-82.6)	63.6 (46.7-82.1)	**-3%**
**Indicator (95%UI)**	**Standardization**	**MALE YEAR 1990**	**2019**	**Change**
Prevalence	All-age	158.2 (145.1-172.7)	229.7 (210.7-250.6)	**45%**
	Age-standardized	142.8 (131.0-155.7)	156.0 (142.6-170.6)	**9%**
Mortality	All-age	0.4 (0.3-0.5)	0.5 (0.4-0.7)	**14%**
	Age-standardized	0.4 (0.3-0.5)	0.3 (0.2-0.4)	**-31%**
YLLs	All-age	8.7 (5.8-10.3)	8.7 (6.6-12.0)	**0%**
	Age-standardized	8.0 (5.3-9.5)	5.2 (4.0-7.2)	**-36%**
YLDs	All-age	20.9 (14.3-28.2)	29.9 (20.6-40.3)	**43%**
	Age-standardized	18.8 (12.9-25.4)	20.6 (14.2-27.9)	**6%**
DALYs	All-age	29.6 (22.8-37.1)	38.6 (28.8-49.2)	**30%**
	Age-standardized	26.8 (20.7-33.7)	25.7 (19.2-32.9)	**-4%**

UI: uncertainty intervals; SDI: socio-demographic index; YLLs: years of life lost, YLDs: years lived with disability; DALYs: disability-adjusted life years

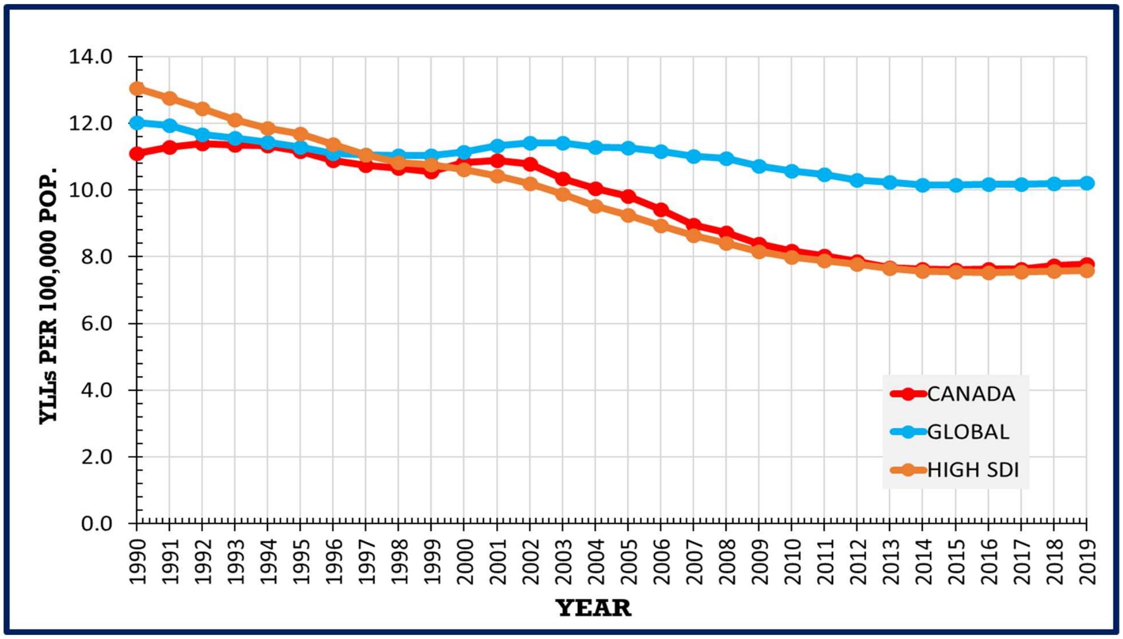

Figure S1. Age-standardized RA YLLs, Canada, global, high SDI, 1990-2019
RA: rheumatoid arthritis; SDI: socio-demographic index; YLLs: years of life lost

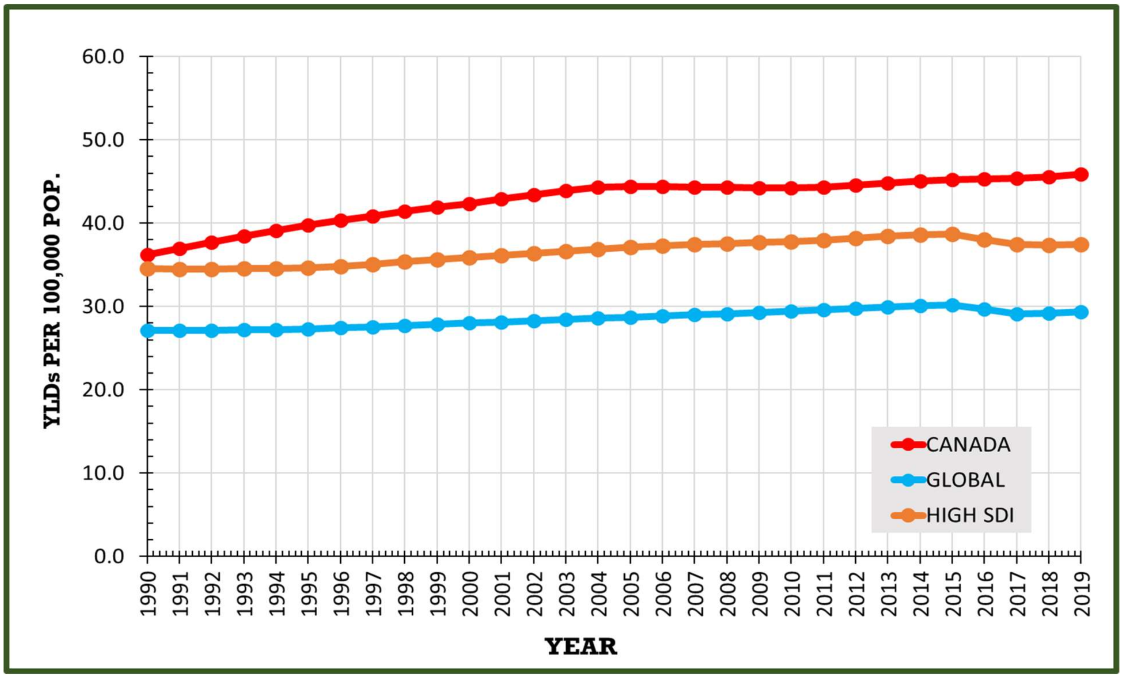

Figure S2. Age-standardized RA YLDs, Canada, global, high SDI, 1990-2019
RA: rheumatoid arthritis; SDI: socio-demographic index; YLDs: years lived with disability

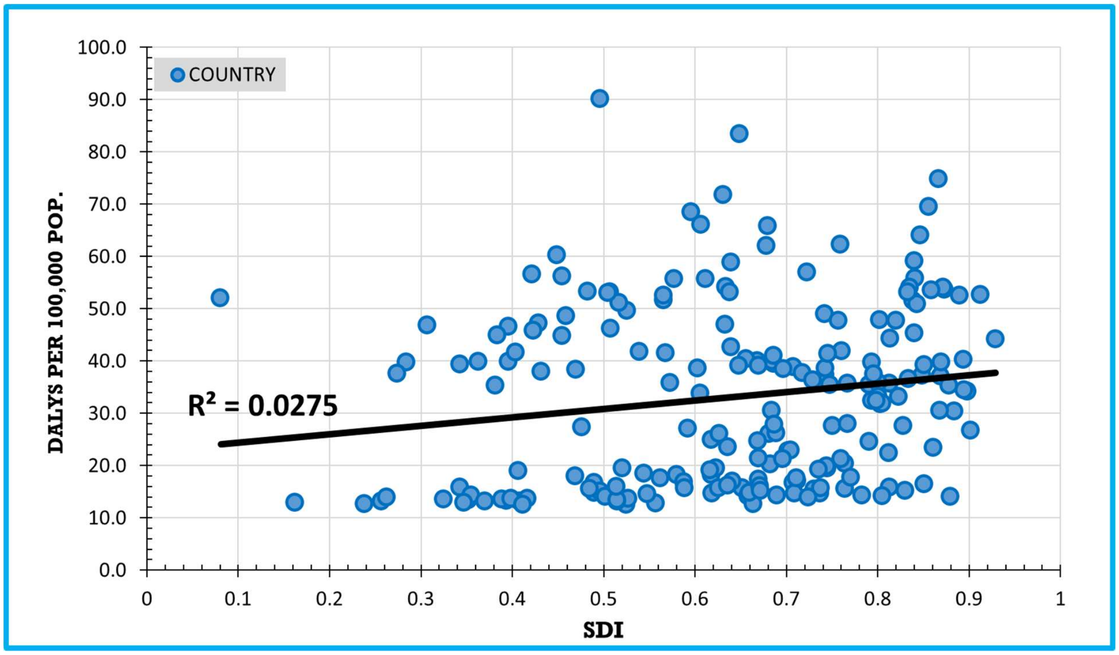

Figure S3. Age-standardized RA DALYs per SDI for 204 countries, 2019

RA: rheumatoid arthritis; SDI: socio-demographic index; DALYs: disability-adjusted life years
